# Self-regulated learning: A key factor in the effectiveness of online learning for second language learners

**DOI:** 10.3389/fpsyg.2022.1051349

**Published:** 2023-01-12

**Authors:** Bo Yu

**Affiliations:** International College of Chinese Language and Culture, Chongqing Normal University, Chongqing, China

**Keywords:** self-regulated learning, influencing factors, second language learners, online learning, learning efficiency

## Abstract

With the further development of Internet technology, online learning has become an important way for learners in the digital age. As an important learning strategy, self-regulated learning plays an important role in e-learning. Whether learners can succeed in the network learning environment largely depends on their online self-regulating learning ability. This research reviews the theories and models of self-regulated learning, and analyzes the influences of individual factors and external factors on second language self-regulated learning in the online learning environment. And some implications in learning and teaching are given, as well as suggestions for future research.

## 1. Introduction

Online learning has become an important way for people to learn in the digital age, thanks to the development of intelligent technologies such as the Internet and the popularization of distance education. Because of the COVID-19 epidemic, online language learning has become the new normal in recent years. Online language learning is a highly autonomous learning mode, with a significantly higher drop-out rate than face-to-face courses. One of the key reasons is that online students lack the ability to self-regulate their learning (Kulusaklı, 2022). As there are differences between online language learning and traditional in-class learning, self-regulation in the context of online foreign language education needs to be measured separately (Wang and Zhan, 2020).

Self-regulated learning (SRL) is an important factor in determining the effectiveness of online language learning for language learners (Cho and Jonassen, 2009; Lin et al., 2021). Paying attention to SRL is beneficial for allowing learners’ subjectivity to be fully expressed in the learning process, as well as for assisting them in developing good learning habits in language learning. When compared to traditional classroom learning, online learning requires more autonomy from students. The online platform, on the other hand, can record learners’ traces (such as learning content and duration) and provide accurate feedback information for SRL. As a result, understanding learners’ self-regulation in second language online learning is critical. SRL is associated with learning and academic achievement, and can guide learners’ future paths to study and work (Viberg et al., 2020). SRL can be trained (Raaijmakers et al., 2018) and controlled by the learners (Chang et al., 2018). Therefore, it is necessary for teachers to provide students with SRL support.

This research will conduct a brief review of the existing literature, mainly discusses two aspects: one is the theory and model of SRL, the other is about the influence factors of SRL on second language online. Finally, based on the above two aspects, also puts forward some implications and suggestions.

## 2. Review of the literature

### 2.1. Theories and models of self-regulated learning

The study of self-regulation originated from the social cognitive theory represented by Bandura (1997). Zimmerman proposed the concept of “self-regulated learning” (SRL) for the first time in Contemporary Educational Psychology in 1986. Since then, educational psychology researchers have explained this concept from various perspectives. Self-regulated learning, according to researchers, occurs when learners take the initiative to set learning goals and then monitor, adjust, and evaluate their cognition, motivation, emotion, behavior, and environment in order to achieve them (Zimmerman and Schunk, 1989; Schunk and Zimmerman, 1994). Despite the fact that its definitions vary, they all emphasize the importance of learners’ initiative and goal setting.

Self-regulated learning is an important concept in the field of learning psychology, especially in the field of learning strategy research, and a core conceptual framework for understanding the cognitive, motivational, and emotional aspects of learning. Researchers have analyzed SRL from different perspectives. Zimmerman (1989) believed that SRL is a process in which learners actively participate in their own learning activities from metacognitive, motivational, and behavioral aspects in order to achieve learning goals. Winne (1995) also believed that language learning is a process of learners’ internal construction and self-orientation. Pintrich (2000) explained more specifically: SRL is an active and systematic learning process in which learners first define their learning goals and then make adjustments according to specific goals to control and monitor their cognition, motivation, and behavior. Numerous studies have shown that effective learning regulation is essential for success (Lawson et al., 2019). SRL is a self-directed process for learners to transform their mental abilities into academic skills. In other words, SRL is the process of helping students manage their thoughts, behaviors, and emotions in order to successfully direct their learning experience (An et al., 2021).

The emergence and development of SRL involve different fields. In the field of educational psychology, researchers apply SRL to different environments for analysis, such as classroom learning (Pionera et al., 2020; Robbins et al., 2020), higher education (Wolters, 1998; Lee, 2002; Fukuda, 2019; Yüce, 2019), and network environment (Azevedo and Cromley, 2004; Gravill and Compeau, 2008; Puntularb et al., 2021; Gambo and Shakir, 2022). Most researchers believe that in order to promote the development of SRL, it is necessary to deeply understand the internal mechanism. How to support and facilitate SRL can be defined only when the relationship between the process and components of SRL is clear.

There are four classical models in the field of educational psychology that describe the process of students’ self-regulating learning. Although all four models believe that SRL is a cyclic process, the stages and focuses of SRL in different models are not consistent. Self-regulation empowerment program model (Zimmerman, 2000) and learning conceptual framework model (Pintrich, 2000) describe the whole process of SRL, and the core components of them are feedback and regulation. Pintrich (2000) also stressed the importance of motivation. Information processing model highlights the role of monitoring at the level of metacognition (Winne and Hadwin, 1998), while dual processing model based on behavior control theory emphasizes the importance of emotion (Boekaerts and Corno, 2005).

Furthermore, new models are constantly being released. The metacognition and emotion model proposed by Efklides (2011), for example, divides SRL into macro and micro levels. Some scholars have tried to integrate new elements into the SRL model, such as integrating epistemic belief (Muis, 2007), epistemic emotion (Muis et al., 2018), and academic emotion (Ben-Eliyahu, 2019).

Language learning strategies have a significant impact on SRL in second language learning (Rose et al., 2018). Dörnyei (2005) pioneered the incorporation of SRL into the field of second language teaching. He emphasized that the definition of language learning strategies was debatable and that the measurement method was not rigorous. His model distinguishes five types of SRL ability: commitment control, metacognitive control, satiation control, emotion control, and environmental control. Based on this model, Tseng et al. (2006) created the questionnaire “Self-regulation ability of vocabulary learning,” which aims to break down the barriers of traditional language learning strategies and measure students’ self-regulation ability to use strategies rather than specific strategies.

In response to criticism of language learning strategies of Dörnyei (2005) and Oxford (2017) redefined and attempted to integrate language learning strategies using SRL and other theories. Language learning strategies, according to her model, are a component of SRL and a means of achieving self-regulation. Although researchers have attempted to propose a SRL model with second language teaching characteristics, its influence remains insufficient. Self-regulation empowerment program model by Zimmerman is still widely used in SRL research in second language teaching. When the models in the two fields are compared, it is discovered that the models in educational psychology have broader and more complete dimensions, richer theoretical perspectives, and are constantly innovating and developing, whereas the models in second language teaching are not only few in number, but also focus on language learning strategies.

In this study, online learning refers to how learners systematically learn a second language through the network using computers and mobile phones in and out of class. Learners’ autonomy in an online learning environment is greater than in an offline learning environment. According to some researchers, self-regulation in online learning differs from that in traditional learning environments (Barnard et al., 2009). Because SRL is a key skill that influences the success of computer-assisted learning (Adeyinka and Mutula, 2010), it is essential to research self-regulation in online learning. Self-regulation in second language online learning can be defined as a self-guided process that allows second language learners to activate and maintain cognition, emotion, and behavior while learning a second language online. SRL in second language online learning shares characteristics of the general self-regulation learning process as well as second language acquisition and the online learning environment. Paying attention to SRL in second language online learning is critical for understanding second language learners’ psychological, cognitive, and behavioral processes, as well as assisting learners in improving their second language level and autonomous learning ability through the use of online learning resources and tools.

### 2.2. Factors influencing self-regulated learning in L2 online learning

Academic achievement is positively related to SRL, particularly metacognitive regulating behaviors like monitoring and planning. A good SRL ability also benefits second language learners’ online learning (Dent and Koenka, 2016). There is also a positive correlation between self-regulation ability and language achievement of second language learners in online environment. So, what factors contribute to differences in SRL ability among learners? The internal individual factors of learners, as well as external training and intervention, will be examined.

#### 2.2.1. Individual factors

Individual motivation to learn is a significant influencing factor. Learners with a clear vision for foreign language learning and an interest in the target language culture can self-regulate more effectively. Motivation is positively correlated with SRL and can predict outcomes (Lim and Yeo, 2021). Hromalik and Koszalka (2018) compared the logs of second language learners at various levels and discovered that those who achieve good online learning results have strong intrinsic motivation, particularly personal emotional motivation. They can better manage their time and will consider and adjust their own learning methods as they reflect. Learners with poor learning outcomes exhibit greater practical motivation and obvious procrastination, and they attribute their poor learning outcomes to external factors. Self-regulation and learning motivation reinforce each other. On the one hand, strong learning motivation can promote second language learners’ self-regulation ability; on the other hand, improving SRL ability also helps to improve learning motivation. When learners have both mastery learning goals dominated by internal motivation and achievement learning goals dominated by external motivation, they typically perform relatively well academically.

Another major influence factor is the language proficiency of the learners. Learners who have been studying foreign languages for a long time will be more inclined to use technology for learning as their language level improves. Research on middle school students’ mobile English vocabulary learning of Liang (2016), for example, discovered that students with higher English proficiency have higher self-regulation ability in mobile vocabulary learning than students with lower English proficiency, particularly in metacognitive control and boredom control. There is a significant positive correlation between the English vocabulary level of middle school students and the self-regulation ability of mobile vocabulary learning. Some students with low language proficiency are hesitant to use social media for language practice because they are “afraid of making mistakes or not being understood by the other party.” Higher-level learners, on the other hand, look for ways to practice their language after class and will use technology more actively to adjust their language learning and achieve their learning goals. Saito (2020) studied the use of L2 strategies and influencing factors of Japanese college English learners, and the research showed that learners’ SRL ability is related to more use of L2 strategies and higher language level.

In addition, self-efficacy influences SRL. Self-efficacy refers to an individual’s belief in his or her ability to accomplish certain tasks and goals (Bandura, 1997). Learners who have a higher sense of self-efficacy are more likely to engage in self-regulating behaviors and devote themselves to learning (Csizér and Tankó, 2017; Sardegna et al., 2018). The ability of self-assessment predicts learners’ self-efficacy in listening, speaking, and reading skills, the ability of environmental organization affects their speaking and writing efficacy, and the factor of goal setting affects their writing efficacy (Su et al., 2018).

#### 2.2.2. External factors

External factors such as guiding and intervening in the online learning process of second language learners will have an impact on their ability to self-regulate. Students who can self-regulate their learning perform better academically, so assisting students in developing this ability is critical. Studies have proved that providing learners with learning strategy guidance can improve self-regulation and academic performance (Bandalos et al., 2003).

Chang (2007) asked Chinese college students learning English to consciously self-monitor in the form of online guidance, and found that students with monitoring strategy guidance were significantly better than students in the control class in terms of reading comprehension and motivation belief, especially for lower-level learners. Deng (2012) took two college English classes as the experimental class and the control class, and cultivated the students’ self-regulating learning ability in the experimental class from five aspects: learning plan, monitoring of learning process, self-evaluation, motivation orientation and reinforcement, and the ability to regulate learning environment. After three semesters of experimental teaching, the experimental class’s final exam and English proficiency test results were clearly superior to those of the control class. Kondo et al. (2012) compared the experiences of Japanese college students learning English listening and speaking in two ways: one through mobile phone software with SRL guidance function, and the other through SRL in a traditional learning environment. According to the study, learners’ language achievement skills improved more noticeably in a mobile learning environment.

Prompt intervention for learners can significantly improve their SRL ability in problem-solving scenarios. It is an effective intervention method to remind students to use specific SRL strategies that they have mastered but have forgotten to use during the learning process (Bannert and Reimann, 2012). Daumiller and Dresel (2019) investigated the effects of metacognitive cues and motivational cues on SRL in students and discovered that motivational cues are more effective. Whipp and Chiarelli (2004) also pointed out that interventions in motivation, self-efficacy, interest, attribution, teacher support, peer assistance, curriculum design, and other factors can improve learners’ online SRL level and learning performance. Bai et al. (2021) regulated and intervened the English writing situation of primary school students in Hong Kong in an online environment, and the research found that the use frequency of four self-regulated learning strategies of students was significantly increased. Chang et al. (2019) studied primary school students in Taiwan who learned English vocabulary on mobile phones and found that students in the experimental group who used the English vocabulary learning APP with self-directed learning mechanism (evllap-srlm) had significantly better academic performance and motivation than those in the control group.

## 3. Implications and suggestions for further research

Although SRL has been shown to be an important factor influencing online learning, learners are not always able to successfully regulate themselves for a variety of reasons, including a lack of good strategy use, a lack of metacognitive knowledge, or a lack of experience in an online learning environment (Li et al., 2018). Besides, according to the social cognitive perspective, SRL is influenced not only by individuals, but also by the interaction of environment and behavior (Zimmerman, 1989). As a result, how to encourage the development of learners’ SRL ability has emerged as a critical issue in research and practice.

To begin with, the research findings indicate that individual factors of learners have a significant impact on self-regulation. As a result, second language online learners should be aware of their own psychological state, adjust their learning motivation, and maintain positive learning beliefs and self-efficacy. For example, learners should encourage themselves and give positive psychological suggestions, believe in their learning ability, and reduce the negative impact of the change of learning style on self-efficacy. Second language learning is a long-term process that requires more self-monitoring and self-regulation from learners. Learners should use goal setting to maintain learning motivation, improve online SRL level, and then improve online language learning effectiveness.

Second, previous research has demonstrated that external intervention and training can significantly improve SRL ability. Without teacher support in teaching, students may overestimate their ability to understand learning materials (Baars et al., 2018), which may have a negative impact on the subsequent learning process. Teachers should provide targeted training and intervention to students, such as combining classroom teaching activities with online learning practice, strengthening SRL strategy training, increasing students’ awareness of SRL strategies, improving effectiveness, assisting students in developing a clear vision of second language online learning, and encouraging and stimulating students’ cultural interest (Zheng et al., 2018).

Last but not least, teachers should pay attention to the evaluation and feedback of students’ self-regulation abilities, as well as recommend more self-regulation resources and methods to students. Teachers can introduce some improvement methods to primary language learners, such as website tools for adjusting speech speed, to help learners make full and efficient use of learning resources (Lai and Gu, 2011). Teachers can also ask students to make more self-observation, self-assessment, and reflection on their online learning process, and conduct detailed analysis and feedback on the results of students’ reflection, so as to help students improve their ability to self-regulate learning.

Based on the above analysis, future research should be carried out from the following aspects: In terms of research content, it is necessary to strengthen the research on the characteristics of online second language learning and SRL mechanism. The forms of second language online learning have become more diverse as educational technology has advanced. The key factors influencing learners’ second language acquisition may differ across learning environments and forms. To find and solve problems in the self-regulation process, an organic combination of SRL theory, language teaching process, and language learning mechanism research is required. Researchers should conduct more specific empirical research, thoroughly investigate the self-regulation process and related influencing factors in second language online learning, and then provide learners with support. In terms of research application, the transformation from research conclusions to teaching application should be enhanced, especially the use of technology to enhance SRL in the online environment, develop more technical tools to support learners to self-regulation, and carry out action research and design-based research in teaching for continuous optimization.

## Author contributions

The author confirms being the sole contributor of this work and has approved it for publication.

## Funding

This study is supported by Center for Language Education and Cooperation, Ministry of Education of the People’s Republic of China, under grant No. 22YH15C and the Education Science Project of Chongqing Academy of Education Science under grant No. 2021-GX-016.

## Conflict of interest

The author declares that the research was conducted in the absence of any commercial or financial relationships that could be construed as a potential conflict of interest.

## Publisher’s note

All claims expressed in this article are solely those of the authors and do not necessarily represent those of their affiliated organizations, or those of the publisher, the editors and the reviewers. Any product that may be evaluated in this article, or claim that may be made by its manufacturer, is not guaranteed or endorsed by the publisher.
